# Farber disease: clinical presentation, pathogenesis and a new approach to treatment

**DOI:** 10.1186/1546-0096-5-15

**Published:** 2007-06-29

**Authors:** Karoline Ehlert, Michael Frosch, Natalja Fehse, Axel Zander, Johannes Roth, Josef Vormoor

**Affiliations:** 1 University Children's Hospital Muenster, Department of Pediatric Hematology and Oncology, Albert-Schweitzer-Strasse 33, D-48149 Muenster, Germany; 2 University Children's Hospital Muenster, Department of General Pediatrics, Albert-Schweitzer-Strasse 33, D-48149 Muenster, Germany; 3 University Hospital Hamburg-Eppendorf, Interdisciplinary Clinic and Policlinic for Stem Cell Transplantation, Martinistrasse 52, D-20246 Hamburg, Germany; 4 Newcastle University, Northern Institute for Cancer Research, Framington Place, Newcastle upon Tyne, NE2 4HH, UK

## Abstract

**Background:**

Farber Disease is an autosomal-recessively inherited, lysosomal storage disorder caused by acid ceramidase deficiency and associated with distinct clinical phenotypes. Children with significant neurological involvement usually die early in infancy, whereas patients without or only mild neurological findings suffer from progressive joint deformation and contractures, subcutaneous nodules, inflammatory, periarticular granulomas, a hoarse voice and finally respiratory insufficiency caused by granuloma formation in the respiratory tract and interstitial pneumonitis leading to death in the third or fourth decade of live. As the inflammatory component of this disorder is caused by some kind of leukocyte dysregulation, allogeneic hematopoietic stem cell transplantation can restore a healthy immune system and thus may provide a curative option in Farber Disease patients without neurological involvement. Previous stem cell transplantations in two children with severe neurological involvement had resulted in a disappointing outcome, as both patients died of progressive deterioration of their neurological status. As a consequence, stem cell transplantation does not appear to be able to abolish or even reduce the neurotoxic effects of the abundant ceramide storage in the brain.

**Methods:**

After myeloablative, busulfan-based preparative regimens, four Farber Disease patients without neurological involvement received an allogeneic hematopoietic stem cell transplantation from related and unrelated donors. Stem cell source was BM in three patients and PBSC in one patient; GvHD-prophylaxis consisted of CsA and short course MTX.

**Results and discussion:**

In all patients, HSCT resulted in almost complete resolution of granulomas and joint contractures, considerable improvement of mobility and joint motility without relevant therapy-related morbidities. All patients are alive and well at this point with stabile donor cell chimerism and without evidence of chronic GvHD or other late sequelae of stem cell transplantation.

**Conclusion:**

Allogeneic hematopoietic stem cell transplantation provides a promising approach for Farber Disease patients without neurological involvement.

## Part I: Clinical presentation of Farber Disease

### Background: First description of Farber Disease

Ceramidase deficiency (Farber lipogranulomatosis or Farber Disease), first described as an inborn storage disease by Farber and coworkers [1,2], leads to tissue accumulation of ceramide due to deficient activity of lysosomal ceramidase.

The clinical presentation of Farber Disease (FD) is characterized by the appearance of subcutaneous skin nodules, ordinarily near the joints, most often interphalangeal, wrist, elbow and ankle joints, or over points of mechanical pressure. These manifestations are very painful and lead to progressive joint stiffness, limitation of motion by contractures and finally to immobilization and deformation of joints. Also, a characteristic sign of FD is the development of a progressive hoarseness due to laryngeal involvement [4].

Beside these major manifestations seven phenotypes have been described which differ in severity and additional organ involvement, like the lungs, nervous system, heart and lymph nodes [4]. Dependent on residual lysosomal ceramidase turnover, patients have a variable degree of central nervous system disease, leading to progressive neurologic deterioration. In most cases the neuronal dysfunction rather than the general physical dystrophy seems to limit the duration of FD [3]. As well, patients with FD may die due to pulmonary disease with interstitial pneumonia.

First symptoms usually appear between the newborn period and the first birthday. Milder forms of type 3 were described with onset at 20 months of age. Clinical manifestation in type 5 of FD, dominated by neurologic deterioration, begins at 1 to 2 1/2 years of life. Patients mainly die within the first years of life, but prolonged courses in patients without severe CNS disease may also be observed [5].

### Phenotypes of Farber Disease

Type 1 is the classic form of the disease with early subcutaneous nodules, joint involvement and hoarseness in all cases. Progressive neurologic involvement and lung disease are reported in many cases [4].

In contrast, type 2 and 3 patients show only slight or no symptoms of central nervous system disease. However, they still have a severe disease as a result of granulomatous inflammation leading to subcutaneous nodules, joint pain and contractures, hoarseness, failure to thrive and respiratory involvement [4].

Patients with type 4 FD present with severe neurologic deterioration and large hepatosplenomegaly already in the neonatal period. Histopathology shows massive granulomatous infiltrations by accumulating macrophages in liver, spleen, lymphoid tissue, thymus and lungs [6].

The major clinical presentation in type 5 patients is a progressive CNS dysfunction, beginning at 1 to 2 1/2 years of life and manifestating in tetraplegia, loss of speech, myoclonia, seizures and mental retardation [7].

Type 6 is a combination of type 1 FD and Sandhoff disease [8], another lysosomal storage disorder caused by hexosaminidase A and B enzyme defects. Both acid ceramidase and hexosaminidase A and B are involved in the catabolism of glycosphingolipids.

One patient is classified as type 7, showing a combined deficiency of glucocerebrosidase, galactocerebrosidase and ceramidase due to a mutation of prosaposin, the precursor protein for two sphingolipid activator proteins [9].

### Diagnosis of Farber Disease

In typical cases of type 1 FD the clinical triad of subcutaneous nodules, joint and laryngeal involvement verifies the disease. When typical features are missing, diagnosis is confirmed by determination of acid ceramidase activity, which is less than 6 percent of control values, measured in cultured skin fibroblasts, white blood cells or amniocytes [4]. Another diagnostic approach is the demonstration of typical histopathologic features on biopsy, showing granulomas with macrophages containing lipid cytoplasmic inclusions in subcutaneous nodules or other tissues [2]. Determination of ceramide accumulation in tissues by chromatography or mass spectrometry is also an established diagnostic test for FD [4].

## Part II: Etiology and pathogenesis of Farber Disease

The curative effect of HSCT on the inflammatory symptoms in FD indicates that the granulomatous inflammation in these patients is not a consequence of mere ceramide storage but reflects a dysregulation of leukocyte functions, probably due to the intracellular role of ceramide in intracellular signal transduction [16]. However, the sequence of molecular mechanisms leading from a defect in ceramide metabolism to chronic granulomatous inflammation still needs elucidation. Alterations of receptor-mediated apoptosis by ceramide accumulation in inflammatory cells may be one of the mechanisms underlying abnormal granuloma formation.

Recent evidence suggests that the sphingolipid ceramide plays an important role as a second messenger in a number of signal transduction pathways. Many cellular responses to extracellular stimuli have been linked to the intracellular generation of ceramide, especially the induction of apoptotic cell death triggered by various stress agents. Ceramide has been proposed to mediate apoptosis, because many pro-apoptotic stimuli induce the production of ceramide and treatment with exogenously added ceramides can induce apoptotic cell death [14].

In a previous study we have shown that cells obtained from a FD patient showed remarkable changes in their sensitivity to different apoptotic stimuli [15]. When treated with staurosporine, chemotherapeutic drugs or ionizing radiation, FD cells underwent apoptosis and activated caspases comparable to control cells. However, due to the lack of ceramidase, cell-permeable ceramides had a stronger pro-apoptotic activity in Farber cells than in controls. Interestingly, we consistently observed an accelerated rate of lymphocyte death in FD upon activation of the death receptor molecule fas (CD95). These data suggest that ceramide does not play an essential role as a general trigger or second messenger in all kinds of apoptosis, but may rather act as a specific amplifier of receptor-induced cell death [15]. There is growing evidence that generation of ceramide increases membrane fluidity and raft formation in the plasma membrane, thereby facilitating receptor mediated signalling. Indeed, recent studies showed that ceramide may be critically involved in cap formation, clustering, and activation of the CD95 receptor [12,13].

As a major symptom FD patients exhibit chronic destructive joint inflammation resembling rheumatoid arthritis. Indeed, increased CD95 receptor/ligand interaction has been implicated in the pathogenesis of inflammatory arthritis [10,11]. It may therefore be speculated that increased CD95 signalling mediated by elevated ceramide levels is involved in the inflammatory arthritis of FD.

## Part III: Therapeutic approaches to Farber disease patients without CNS involvement

### Introduction

For some decades now, allogeneic hematopoeitic stem cell transplantation has proven to provide a potential, curative approach to malignant [17] and non-malignant diseases. Additionally, it has also shown efficacy in some inherited disorders, predominantly primary immunodeficiencies and lysosomal storage disorders [19,24]. Symptoms of disease in these metabolic disorders are usually caused by the lack of enzyme activity. The transplantation of hematopoetic stem cells from a healthy donor can be a source of a sufficient amount of enzyme and thus abolish or at least decrease or stabilize symptoms of disease in some patients [18,20-22].

The etiology of Farber disease is the lack of acid ceramidase, and subsequently there is an increased storage of ceramide in several organs and tissues [4]. However, as already illuminated in the previous two chapters of this article, the main symptoms of disease in these patients, at least in those without involvement of the central nervous system, are caused by some kind of leukocyte dysregulation [15]. The dysfunction of the immune system results in the inflammatory component of the disorder with painful swelling of several joints, granuloma formation, contractures and inflammatory airway involvement. Current management for these patients has therefore focused on pain therapy, physcial therapy, surgical correction of severe contractures and anti-inflammatory medication. The affected patients usually die in the second decade of their lives as a consequence of granuloma formation in the respiratory tract and their immobility with recurrent episodes of pneumonia and resulting respiratory insufficiency [4].

### Previous reports on allogeneic HSCT in Farber disease patients

The first reports on hematopoietic stem cell transplantion in two Farber disease patients were published in 1989 and 2000 by Souillet and Yeager [25,26]. However, both children had a subtype of Farber disease with involvement of the central nervous system and died because of a deterioration of their neurological status. So, as is also the case in other lysosomal storage disorders with severe CNS disease [23], hematopoietic stem cell transplantation does not seem to provide a benefit for these children, as it is not able to abolish or even reduce the neurotoxic effects of excessive ceramide accumulation in the brain. However, as HSCT can provide a healthy immune system and thus correct the abnormal inflammatory response and symptoms of disease, there could be an option for hematopoietic stem cell transplantation in those children with a predominance of the inflammatory component of the disorder. Additionally, resolution of granulomas after hematopoietic stem cell transplantation could also be demonstrated in children with central nervous system involvement [25,26].

### Present experience of allogeneic HSCT in Farber disease patients without CNS involvement

Starting in 2001, three patients in Muenster [27] and one patient in Hamburg have been transplanted so far. Diagnosis was confirmed by measurement of the activity of acid ceramidase in fibroblast cultures (patients #1, #2 and #3 in Toulouse, France, and patient #4 in Baltimore, USA). All patiens had the typical findings of Farber disease with granuloma formation, a hoarse voice, painful swelling of their joints with partially severe contractures and restriction of mobility. Table 1 shows the transplant related data of these patients.

**Table 1:** Transplant-related data of patients

**Parameter**	**Patients**			
**Patient number and gender**	**#1, female**	**#2, male**	**#3, female**	**#4, female**

**Patient origin**	Muenster	Muenster	Muenster	Hamburg
**Age at SCT**	3 years	3 years	2 years	21 years
**Preparative regimen**	Bu/Cy	Bu/Cy/ATG	Bu/Cy	Bu/Cy/ATG
**Stem cell source and dose (no T-cell depletion)**	BM (MRD), 11.3 × 10^6 ^CD34	PBSC (MUD), 25 × 10^6 ^CD34	BM (MRD), 5.6 × 10^6 ^CD34	BM (MRD), 4.2 × 10^6 ^CD34
**GvHD prophylaxis**	CsA, MTX	CsA, MTX	CsA, MTX	CsA, MTX
**Neutrophil engraftment**	day +14	day +12	day +18	day +14
**Acute/chronic GvHD**	grade II of gut/none	grade I of gut/none	grade II of skin/none	none
**Therapy of acute/chronic GvHD**	steroids/none	steroids/none	steroids + tacrolimus/none	none
**Infections**	Clostridium difficile enteritis, bacteremia with Staphylococci	CMV reactivation	CMV colitis	none
**Toxicities**	CsA-neurotoxicity	None	CsA-neurotoxicity	suspected VOD
**Current donor cell chimerism**	91%	95%	90%	99%

The preparative regimen, consisting of intravenous (patients #1 to #3) or oral busulfan (patient #4) and cyclophosphamide, was tolerated well; no neurological, renal, skin or lung toxicities were observed. In patients #1, #3 and #4 bone marrow was the stem cell source, in patient #2 peripheral blood stem cells were used; no in-vitro-T cell depletion was performed. GvHD prophylaxis consisted of ciclosporine and short course methotrexate. Engraftment was noted between days +12 and +18. Patients #1, #2 and #3 developed mild acute GvHD, but no evidence of chronic GvHD so far. Therapy for acute GvHD consisted of steroids in all affected patients, and in patient #3, of additional tacrolimus instead of ciclosporine, as she experienced ciclosporine-induced-neurotoxicity. Transplant related morbidity in our patients was moderate. CMV-reactivation and -colitis in patients #2 and #3, respectively, was controlled by intravenous ganciclovir. For treatment of Clostridium difficile enteritis and Staphylococci bacteremia, patient #1 was given oral vancomycin and iv teicoplanin. On routine ophthalmological examination on day +100, patient #1 was diagnosed with papillary edema, most probably due to elevated intracranial pressure as a consequence of medication with ciclosporine; she was switched to tacrolimus, although she did not suffer from clinical neurotoxicity. In contrast, clinical signs of neurotoxicity by ciclosporine were observed in patient #3; she presented with a generalized seizure, stroke-like symptoms and the characteristic findings in MR imaging studies. Both patients #1 and #3 were switched from ciclosporine to tacrolimus without further difficulties. Hemiparesis in patient #3 gradually resolved within a few months.

**Figure 1:**
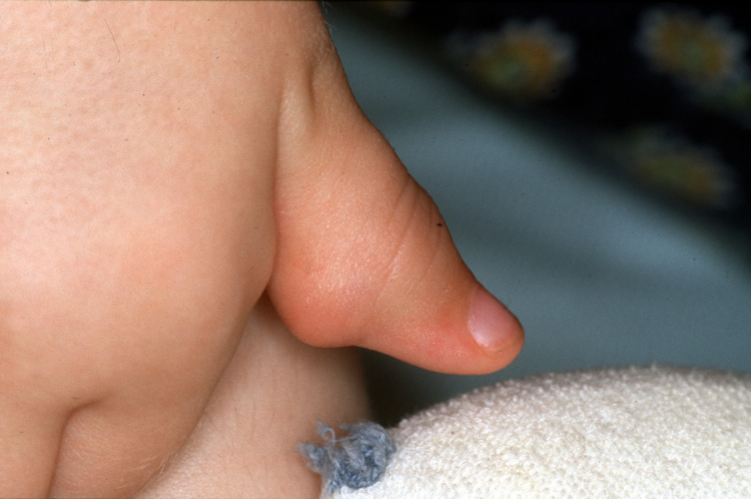
Pre-transplant status of patient #1, right hand (thumb), with inflammatory nodules.

**Figure 2:**
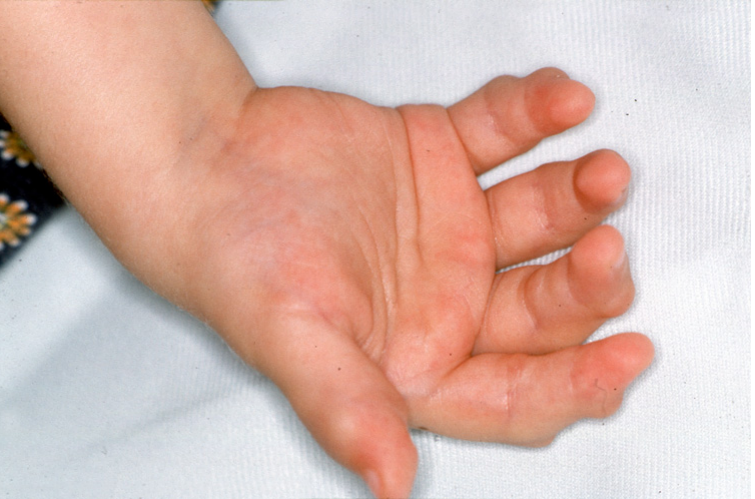
Pre-transplant status of patient #1, right hand (palmar view), with identical findings as described in Figure 1.

**Figure 3:**
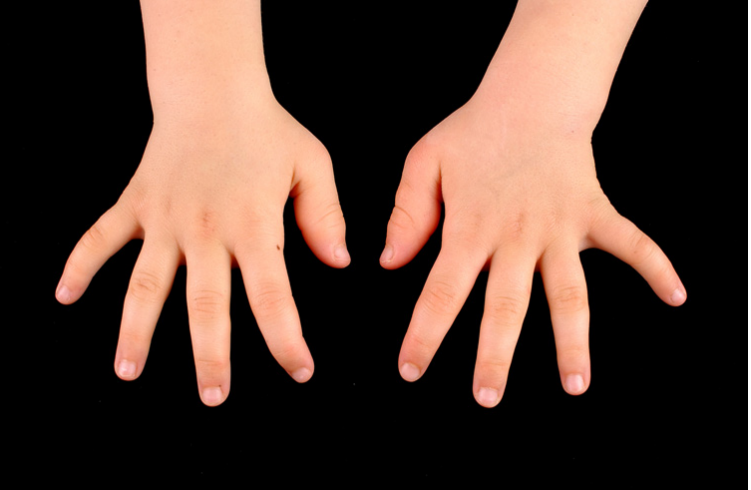
18 months post-transplant status of patient #1 with complete resolution of inflammatory nodules.

In contrast to the natural course of disease in Farber patients, which is characterized by progressive joint deformation, immobility, pain and finally restriction to wheel-chairs, all of the transplanted patients demonstrated a gradual improvement in mobility, reduced number of joints with restricted motion, less pain and significant gain of function. Prior to her transplant, patient #1 was almost wheel-chair bound and could hardly walk, as she suffered from severe contractures in numerous joints. Figures 1 to 3 clearly illustrate the substantial benefit of HSCT in this patient, as shown with the pre- and post-transplant status of her hands.

Patient #4 was a young woman at the time of her transplant, having a milder course of disease compared to the other patients, which might reflect a different subtype on a molecular basis. The reason for HSCT was the need of numerous surgical procedures in her legs to improve mobility. Currently, all patients are seen at least twice yearly by the outpatient BMT unit; additionally, all children are evaluated by pediatric rheumatology once yearly. Inflammatory markers (ESR, leukocyte count, CRP) are checked regularly, however, no data is available with regard to specific granulocyte activation markers. Table 2 summarizes the findings in the post-transplant course of our patients.

**Table 2:** Post-transplant follow-up of patients

**Parameter**	**Patients**							
**Patient number and gender**	**#1, female**		**#2, male**		**#3, female**		**#4, female**	

**Patient origin**	Muenster		Muenster		Muenster		Hamburg	

**Time point of follow-up**	**prior to SCT**	**day +450**	**prior to SCT**	**day +200**	**prior to SCT**	**day +270**	**prior to SCT**	**day +958**

**ESR (mm, first hour, Westergren)**	38	9	53	12	25	13	no data available	
								
**Time point of follow-up**	**prior to SCT**	**day +1800**	**prior to SCT**	**day +1560**	**prior to SCT**	**day +990**	**prior to SCT**	**day +958**

**Number of nodules**	58	0	39	0	18	0	pre Tx-nodules mainly dissolved, no new nodules	
**Number of joints with restricted mobility**	26	0	24	0	10	0	almost all joints affected, however considerable improvement after HSCT	

Legend: The clinical evaluation of patients has been followed more closely, so laboratory data (i.e. ESR) are not available at each time-point of follow-up.

### Summary

It is clearly evident that the present experience with hematopoietic stem cell transplantation in Farber Disease is still limited. HSCT may not be appropriate for patients with CNS involvement as ceramide neurotoxicity may not be reversible by stem cell transplantation. After a myeloablative, busulfan-based preparative regimen, a rapid engraftment was demonstrated with only moderate acute GvHD, no signs of chronic GvHD and low therapy-related morbidity. Donor cell chimerism is between 90 and 100% which seems to be sufficient for important reduction of disease-specific symptoms as all transplanted patients had a considerable improvement in joint mobility and a decreasing number of granulomas.

In conclusion, despite the preliminary nature of the data on hematopoietic stem cell transplantation in Farber Disease without central nervous system involvement, we have demonstrated a promising therapeutic approach for these patients.

## Abbreviations

ATG anti-thymocyte globulin

BM bone marrow

BU busulfan

CD cluster of differentiation

CMV cytomegalovirus

CNS central nervous system

CRP C reactive protein

CsA ciclosporin A

CY cyclophosphamide

ESR erythrocyte sedimentation rate

FD Farber Disease

GIT gastro-intestinal tract

GvHD graft-versus-host-disease

HSCT hematopoietic stem cell transplantation

IV intravenous

MR magnetic resonance

MRD matched related donor

MTX methotrexate

MUD matched unrelated donor

PBSC peripheral blood stem cells

SCT stem cell transplantation

Tx transplantation

VOD veno-occlusive disease

## Competing interests

The author(s) declare that they have no competing interests.

## Authors' contributions

The concept of allogeneic stem cell transplantation in these children was developed by Josef Vormoor and Johannes Roth. Michael Frosch describes the clinical phenotypes of FD, Johannes Roth has written the part on etiology and pathogenesis, Karoline Ehlert part III on recent experiences with stem cell transplantation. Natalja Fehse and Axel Zander have reported the outcome of their patient transplanted in Hamburg. All authors have read and approved the final manuscript.

## Informed consent

Written informed consent for the publication of the photographs has been obtained by the patients respectively their parents.
